# Conservation of Terrestrial Vertebrates in a Global Hotspot of Karst Area in Southwestern China

**DOI:** 10.1038/srep25717

**Published:** 2016-05-26

**Authors:** Zhenhua Luo, Songhua Tang, Zhigang Jiang, Jing Chen, Hongxia Fang, Chunwang Li

**Affiliations:** 1 Key Laboratory of Animal Ecology and Conservation Biology, Institute of Zoology, Chinese Academy of Sciences, Beijing 100101, China; 2 School of Life Sciences, Central China Normal University, Wuhan 430079, China; 3 University of Chinese Academy of Sciences, Beijing 100049, China

## Abstract

The karst area of southwest China (KASC) is the largest piece of karst landscape on the earth and a global biodiversity hot-spot with high concentrations of endemic species. Although a number of nature reserves (NRs) have been established across the region, the representativeness of biodiversity of the NR system is still unknown. Based on comprehensive literature and field surveys, and intensive consultations with zoologists and wildlife managers, we compiled distributions of 1,204 terrestrial vertebrate species and 271 NRs in KASC. We found Jinxiu, Mengla, Hekou, and Jinghong have the richest amphibian species; Jinxiu has the highest species richness of reptiles; Jinghong, Menghai, and Mengla have the largest numbers of avian species; whereas, Mengla, Longzhou, and Ningming have the greatest mammalian diversity in the region. Gap analysis among NR system, species richness pattern, and five biogeographic indicators found insufficient representation of the NR system on territorial vertebrate diversity. The conservation effectiveness in Guizhou Province was much lower than that in Guangxi and Yunnan Provinces. Under-representation and over-representation simultaneously occurred in many of the ecoregions, elevation classes, vegetation types, landcover categories, and human disturbance intensity gradients. For conservation of terrestrial vertebrates in KASC, several suggestions were presented in this study.

Biodiversity loss is among the most serious environmental problems worldwide, in large part, the problem is attributed to rapid and extensive human-mediated habitat destruction and conversion^1^,^2^,^3^,^4^,^5^,^6^,^7^,^8^. The need to preserve biodiversity is therefore urgent. *In situ* conservation is wildly recognized as a fundamental approach for maintenance and protection of wild animals^2^,^9^,^10^. One of the main actions to design and establish nature reserves (NRs) is to select the localities with high conservation values, which should take into consideration the representation and persistence of biodiversity^9^,^11^,^12^. Thus, spatial pattern of species richness (SR) is a critical issue in biodiversity conservation^13^, which requires comprehensive assessment of species distributions to evaluate the importance of different sites and effectiveness of management activities^10^,^14^,^15^.

Many NR systems do not adequately represent regional biodiversity; not all the NRs are able to meet their conservation goals as a whole, because social-economic and aesthetic criteria usually predominated in the site selection of NR^16^,^17^,^18^. Thus, Gap analysis, which identifies the gaps in protected areas, is a powerful planning approach to explore to what extent animal and plant species are being protected and assesses the effectiveness of a NR system in representing local biodiversity^14^,^19^. Furthermore, conservation planning at landscape scale, based on assessments of the conservation values among categories of different biological and geographical attributes, has particular benefits for conducting Gap analysis of biodiversity conservation, because biogeographical parameters are effective surrogates of other aspects of biodiversity^14^. Although there might be a limitation of no explicit analysis on the connectivity among species distribution ranges, biodiversity hotspots, or NRs^20^, Gap analysis has been applied to various taxa globally^2^,^3^, at continental scale^4^, and in many countries^21^,^22^,^23^,^24^,^25^. Gap analyses at national and regional scales are required to upgrade the conservation efficiency of the NR systems. In addition, there are generally four levels of NRs (national, provincial, municipal NRs and NRs at the county level) in China^26^. So-called “paper NRs” exist as a result of social conflicts, insufficient funding, inadequate capacity for conservation action, or lack of conservation interests by local governments and communities^27^,^28^. Gap analyses on the different levels of NRs could provide empirical data to management effectiveness assessment of Chinese NR system and could give valuable suggestions for the improvement of its management efficiency.

Karst area in southwestern China (KASC) is the largest area of karst landscape on the earth^29^ (Fig. 1). It is one of the 25 global biodiversity hotspots and has a unique biome which contains many endemic and threatened species and high species richness^1^,^30^. Little is known about its species richness pattern and no comprehensive study of biodiversity was carried out in this hotspot. NR system was established in this area over the years^31^, however, no dataset is available for conducting analyses of its conservation efficacy. Thus, a rapid biodiversity assessment and a Gap analysis are urgently needed to guide the conservation strategies in this area and enhance its NR system.

Our aims were to answer these questions: (1) What is the species richness pattern of terrestrial vertebrates in this area? (2) What are the importance of terrestrial vertebrate resources and biodiversity conservation in this area to the conservation efforts at the national scale? (3) Is there any gap of national and provincial NR system in terrestrial vertebrate conservation at county level in this area? For answering above questions and providing suggestions on biodiversity conservation in this area, we compiled the inventory of the terrestrial vertebrates at county level and assessed the values and conservation capacities of terrestrial vertebrate resources in KASC. Based on spatial datasets of distributions of national and provincial NRs and five biogeographic variables (elevation, ecoregion, vegetation type, landcover, and human disturbance), we also carried out Gap analyses to evaluate the conservation efficacy of the NR system.

## Results

### Species Richness Patterns

There are 1,204 terrestrial vertebrate species in KASC, including 180 mammals (30% of the national total), 643 birds (48% of the national total), 205 reptiles (53% of the national total), and 176 amphibians (58% of the national total). Our results showed that the site with the richest amphibian diversity locates at Jinxiu County in Guangxi, followed by Mengla County, Hekou County, and Jinghong City in Yunnan. The rest counties had <70 of amphibian species (Fig. 2). Reptile species richness in Jinxiu County is also the highest in this area, followed by Longzhou County and Nanning City in Guangxi, Mengla County and Jinghong City in Yunnan. The rest counties had <120 of reptile species, with most of them had <100 of reptile species (Fig. 2). Jinghong City, Menghai County, and Mengla County in Yunnan had the greatest bird diversity of >400 species, followed by Kunming City and Mengzi County in Yunnan, Jinxiu County and Longzhou County in Guangxi, where have 250–300 bird species. The rest counties had <250 of bird species (Fig. 2). Mengla County, Longzhou County, and Ningming County in Guangxi had the highest mammalian diversity, with each of them had >100 of mammals species, whereas the rest counties had <70 of mammalian species (Fig. 2).

Twenty-one counties are included in the areas with top 10% species richness of terrestrial vertebrates, including eleven counties in Yunnan, eleven counties in Guangxi, five counties in Guizhou, one county in Sichuan, and one county in Chongqing; all of these counties have >300 terrestrial vertebrate species (Fig. 3). A total of 978 terrestrial vertebrate species occur in these top 10% biodiversity counties and they have a high representativeness (81.2%) of terrestrial vertebrate species in KASC. The 21 counties represent 77.3% of the amphibian species (136 species), 82.4% of the reptile species (169 species), 83.5% of the bird species (537 species), and 75.6% of the mammal species (136 species) that distribute in the karst area.

### Importance of Terrestrial Vertebrates in KASC

One hundred and sixty-one endemic terrestrial vertebrate species (63 amphibian species, 41 reptile species, 39 bird species, and 18 mammal species) were recorded in KASC, accounting for 36%, 20%, 6%, and 10% of the total species numbers of amphibians, reptiles, birds, and mammals in the area, respectively. The importance of terrestrial vertebrate resources and their conservation for KASC were summarized in Table 1.

### NR System

Till the end of 2011, 271 NRs had been established in KASC, with an area of 50,950.85 km^2^ or 9.41% of the total area of the karst region in southwest China. Yunnan established 89 NRs which covered 21,411.65 km^2^, while Guangxi established 51 NRs which covered 13,779.24 km^2^. Thirty-seven, eighty-eight, four, and two NRs, with areas of 8,086.97 km^2^, 5,829.31 km^2^, 1,139.09 km^2^, and 18.95 km^2^, had been set up within the karst landscape in Hunan, Guizhou, Chongqing, and Hubei, respectively. There were 27 national NRs (23,037.33 km^2^; 4.26% of the total area of KASC, Fig. 4) in KASC, including eleven in Yunnan, eight in Guangxi, five in Guizhou, and three in Hunan. Eighty-six provincial NRs (22,037.43 km^2^; 4.08% of the total area of KASC) had been found in this area (Fig. 4). Comparing to the NR system at the end of 1990s (20 national NRs with 12,208.72 km^2^ (2.26% of the total area of KASC) and 65 provincial NRs with 19,581.11 km^2^ (3.62% of the total area of KASC)), NR numbers and total coverage of the NR system were increased by one third and 50%, respectively, and the total area of national NRs was almost doubled (Fig. 4).

### Conservation Gaps

Overlay of the national and provincial NR system and species richness pattern showed that most of the counties with rich animal diversity (top 10% SR) were more or less under the protection of national and provincial NRs (Fig. 3). By contrast, counties with high biodiversity in Guizhou were rarely protected (<1% of the areas of the top 10% SR counties were covered by NRs; Fig. 3). Furthermore, among the counties containing NRs in KASC, eighteen were overlaid with national NRs and seventeen were under the protection of provincial NRs (Fig. 3). Zunyi City, Ruiyang County, and Yixing County in Guizhou are with species richness of 350–400, but no NRs were established there yet (Fig. 3). For Yunnan and Guangxi, Kunming City, Simao County, Mengzi County, Tiane County, Shangsi County, Qinzhou County, and Nanning City were under insufficient protection (<5% of their areas were covered by NRs), while similar situations were emerged in Youyang County of Chongqing and Huidong County of Sichuan (Fig. 3).

Northern Indochina subtropical forest ecoregion has the largest number of terrestrial vertebrate species (928 species), which is followed by Jian Nan subtropical evergreen forest (724 species), southeast China-Vietnam subtropical evergreen forest (657 species), and Yunnan Plateau subtropical evergreen forest ecoregions (633 species) (Fig. 5a). Our results showed a general pattern that the SR decreases with the increase of elevation, and the areas under 1,500 m have great species richness (500–1,000 m: 929 species, 1,000–1,500 m: 928 species, <500 m: 657 species) (Fig. 5b). Among the vegetation types, subtropical acid soil evergreen-broadleaved deciduous shrub forest, coppice and meadow, tropical-subtropical economic forest and orchard, tropical semi-evergreen broadleaf monsoon forest and secondary vegetation, subtropical evergreen broadleaf forest, and tropical evergreen rain forest carry over 800 species (Fig. 5c). For the landcover types, 928 and 878 vertebrate species occur in the mosaic of cropping and forest mosaic/degraded forest areas, and >700 species were recorded in the needleleaved evergreen forests and bush lands (Fig. 5d). The greatest SR were found in the regions with low human disturbance, that is, nearly 1,200 and nearly 1,000 species distribute in the areas with 20–40 and <20 of HFP values, respectively (Fig. 5e).

Seven ecoregions distribute in KASC (Fig. 6a). Among them, the Guizhou Plateau broadleaf and mixed forest ecoregion has the largest coverage (174,540.63 km^2^), followed by the Yunnan Plateau subtropical evergreen forest ecoregion (146,997.74 km^2^) and the Jian Nan subtropical evergreen forest ecoregion (95,264.92 km^2^) (Fig. 6a). The northern Indochina subtropical forest ecoregion and the southeast China-Vietnam subtropical evergreen forest ecoregion have areas of over 50,000 km^2^, respectively (Fig. 6a). Our results showed that all the ecoregions have low proportions of areas (<7%, Fig. 6a) covered by NRs, especially the Qinghai-Minshan conifer forest ecoregion which has only 1.24% of its area was overlain with NR system and whose area designed for conservation remained unchanged during past 20 years (Fig. 6a). The conserved area in Changjiang Plain evergreen forest ecoregion was obviously increased after 1990 (from 0% to 3.42%) and that in Guizhou Plateau broadleaf and mixed forest was nearly tripled (from 2.12% to 6.31%) (Fig. 6a). Most of the conserved areas in Jian Nan subtropical evergreen forest, Guizhou Plateau broadleaf and mixed forest, and northern Indochina subtropical forest ecoregions are covered by national NRs (Fig. 6a).

**Figure 1:**
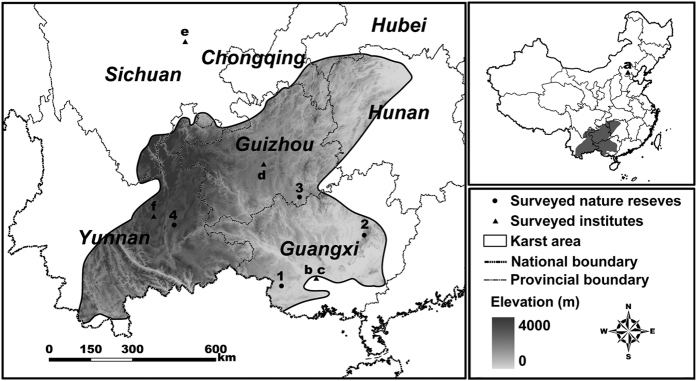
Map of karst area in southwestern China. The labels on the map: (a) Institute of Zoology, CAS; (b) Guangxi Normal University; (c) Guangxi Institute of Biology, (d) Guizhou Institute of Biology; (e) Chengdu Institute of Biology, CAS; (f) Kunming Institute of Zoology, CAS; 1-Nonggang National Nature Reserve; 2-Dayaoshan National Nature Reserve; 3-Maolan National Nature Reserve; 4-Shilin Geological Park and Changhu Park. This figure is generated based on DEM data (CGIAR-CSI) from the International Center for Tropical Agriculture (CITA) and CGIAR International Research Centers (CITA & CGIAR International Research Centers. *The CGIAR consortium for spatial information* (*CGIAR-CSI*). (CITA, Cali, Colombia, 1999). Available at: http://srtm.csi.cgiar.org/) and the karst area boundary was digitized from the Compiling Committee of Comprehensive Physical Regionalization in China using ArcGIS 9.3 (ESRI, Redland, CA; Available at: http://www.esri.com/).

**Figure 2:**
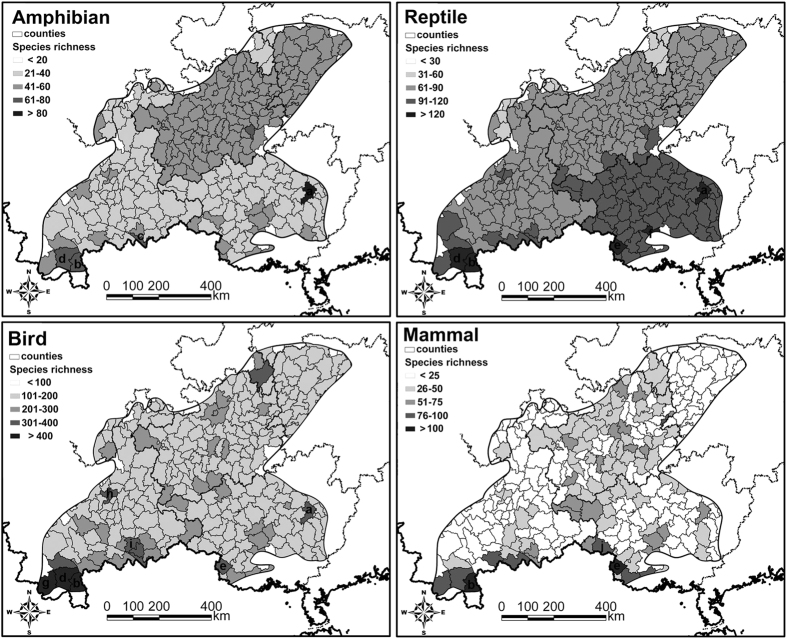
Species richness pattern of amphibians, reptiles, birds, and mammals in the karst area, southwestern China. Labels on the map are: (a) Jinxiu County, (b) Mengla County, (c) Hekou County, (d) Jinghong City, (e) Longzhou County, (f) Nanning City, (g) Menghai County, (h) Kunming City, (i) Mengzi County, (j) Ningming County. These panels are generated based on the number of species for amphibians, reptiles, birds, and mammals within each county and the karst area boundary was digitized from the Compiling Committee of Comprehensive Physical Regionalization in China using ArcGIS 9.3 (ESRI, Redland, CA; Available at: http://www.esri.com/).

**Figure 3:**
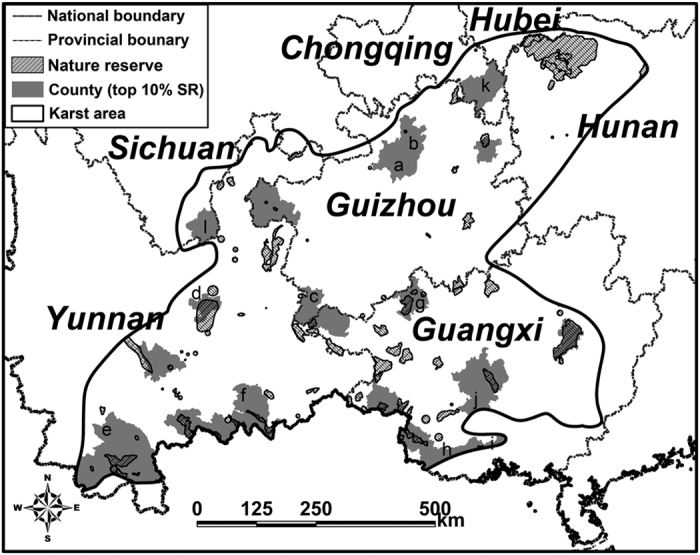
Gap analysis of species richness (SR) pattern and distributions of nature reserves (NRs). Only national and provincial NRs and the counties with top 10% of species richness were showed on the map. Labels on the map are counties: (a) Zunyi City, (b) Ruiyang County, (c) Yixing County, (d) Kunming City, (e) Simao County, (f) Mengzi County, (g) Tiane County, (h) Shangsi County, (i) Qinzhou County, (j) Nanning City, (k) Youyang County, (l) Huidong County. This figure is generated based on species richness for each county, the karst area boundary was digitized from the Compiling Committee of Comprehensive Physical Regionalization in China, and the nature reserve boundaries were from the World Database on Protected Areas (IUCN & UNEP-WCMC. *The World Database on Protected Areas* (*WDPA*) *[Online], [Version 2011.10]*. (UNEP-WCMC, Cambridge, UK, 2011). Available at: http://www.protectedplanet.net/) using ArcGIS 9.3 (ESRI, Redland, CA; Available at: http://www.esri.com/).

**Figure 4:**
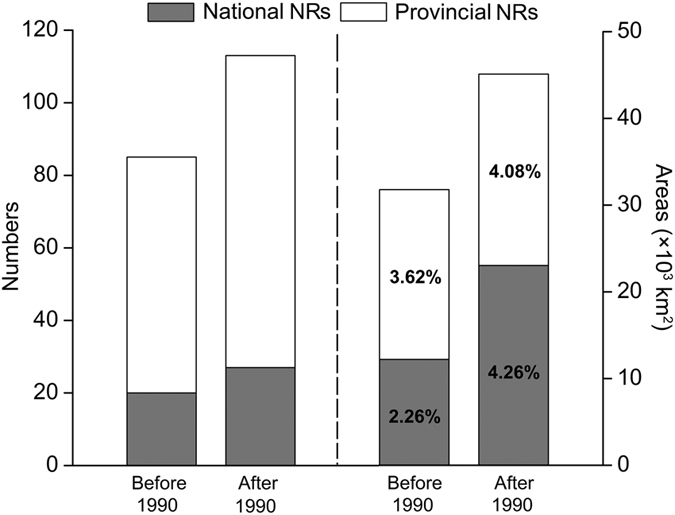
Numbers and areas of national and provincial NRs in the karst area, southwestern China. NRs established before and after 1990 (till 2011) were summarized separately. Statistics in the bars show the percentages of areas covered by NRs to the total area of the karst area, southwest China.

**Figure 5:**
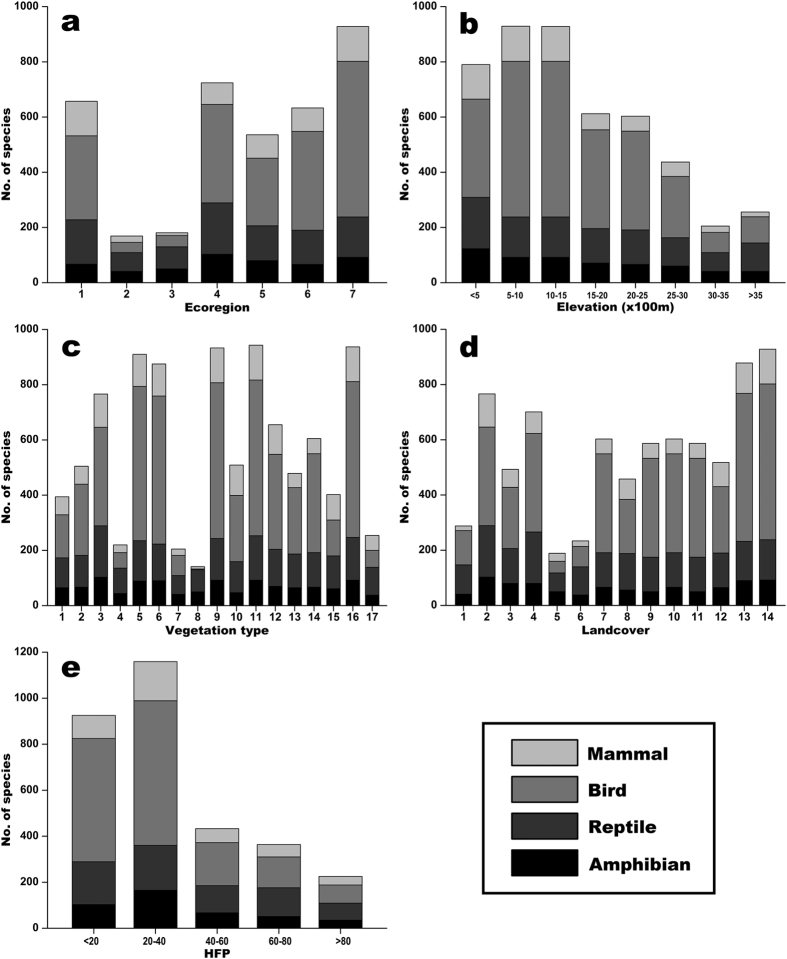
Species richness patterns of terrestrial vertebrates (amphibians, reptiles, birds, and mammals) among ecoregions (**a**), elevation classes (**b**), vegetation types (**c**), landcover categories (**d**), and human footprint (HFP) gradients (**e**). Ecoregion (**a**): 1-southeast China-Vietnam subtropical evergreen forest; 2-Qinghai-Minshan conifer forest; 3-Changjiang Plain evergreen forest; 4-Jian Nan subtropical evergreen forest; 5-Guizhou Plateau broadleaf and mixed forest; 6-Yunnan Plateau subtropical evergreen forest; 7-northern Indochina subtropical forest. Vegetation type (**c**): 1-desert/none vegetation; 2-subtropical-tropical evergreen conifer forest; 3-subtropical limestone broadleaf and mixed forest; 4-subtropical acid yellow soil broadleaf and mixed forest; 5-subtropical evergreen broadleaf forest; 6-tropical evergreen rain forest; 7-subtropical evergreen sclerophyllous broadleaf forest; 8-subtropical bamboo forest; 9-tropical semi-evergreen broadleaf monsoon forest and secondary vegetation; 10-tropical evergreen rain forest and secondary vegetation; 11-subtropical acid soil evergreen-broadleaved deciduous shrub forest, coppice and meadow; 12-tropical-subtropical limestone lianoid vegetation; 13-tropical-subtropical day shrub forest and grassland; 14-subtropical double cropping farmland; 15-tropical-subtropical triple cropping farmland; 16-tropical-subtropical economic forest and orchard; 17-open water. Landcover (**d**): 1-bare land; 2-needleleaved evergreen forest; 3-broadleaved deciduous forest; 4-bush; 5-sparse woods; 6-seaside wetland; 7-alpine and sub-alpine meadow; 8-slope grassland; 9-city and town; 10-river; 11-lake; 12-farmland; 13-Mosaic of cropping; 14-forest mosaic/degraded forest.

**Figure 6:**
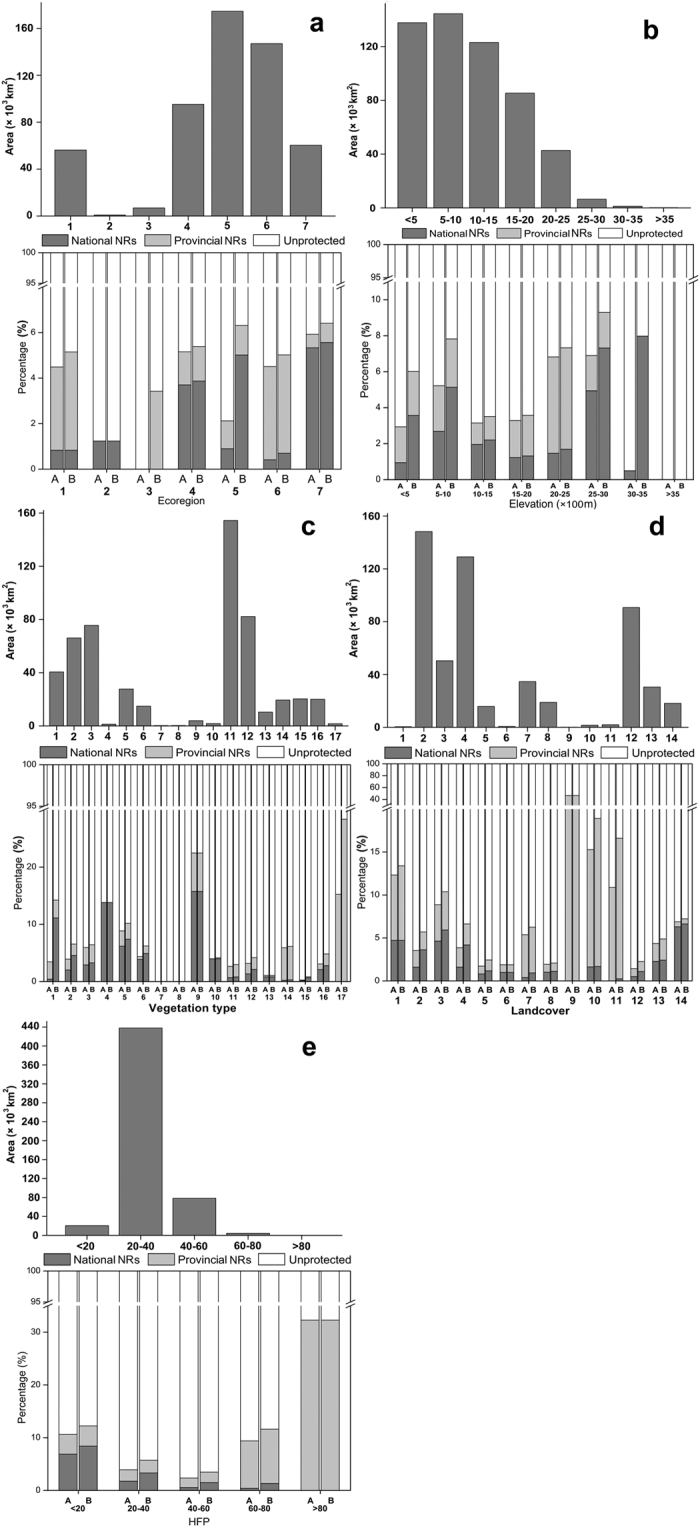
Distributions of areas and representations of national and provincial NRs by ecoregion (**a**), elevation class (**b**), vegetation type (**c**), landcover category (**d**), and human footprint (HFP) gradient (**e**). Labels under the horizontal ordinates: A-before 1990; B-after 1990 (till 2011). Ecoregion (**a**): 1-southeast China-Vietnam subtropical evergreen forest; 2-Qinghai-Minshan conifer forest; 3-Changjiang Plain evergreen forest; 4-Jian Nan subtropical evergreen forest; 5-Guizhou Plateau broadleaf and mixed forest; 6-Yunnan Plateau subtropical evergreen forest; 7-northern Indochina subtropical forest. Vegetation type (**c**): 1-desert/none vegetation; 2-subtropical-tropical evergreen conifer forest; 3-subtropical limestone broadleaf and mixed forest; 4-subtropical acid yellow soil broadleaf and mixed forest; 5-subtropical evergreen broadleaf forest; 6-tropical evergreen rain forest; 7-subtropical evergreen sclerophyllous broadleaf forest; 8-subtropical bamboo forest; 9-tropical semi-evergreen broadleaf monsoon forest and secondary vegetation; 10-tropical evergreen rain forest and secondary vegetation; 11-subtropical acid soil evergreen-broadleaved deciduous shrub forest, coppice and meadow; 12-tropical-subtropical limestone lianoid vegetation; 13-tropical-subtropical day shrub forest and grassland; 14-subtropical double cropping farmland; 15-tropical-subtropical triple cropping farmland; 16-tropical-subtropical economic forest and orchard; 17-open water. Landcover (**d**): 1-bare land; 2-needleleaved evergreen forest; 3-broadleaved deciduous forest; 4-bush; 5-sparse woods; 6-seaside wetland; 7-alpine and sub-alpine meadow; 8-slope grassland; 9-city and town; 10-river; 11-lake; 12-farmland; 13-Mosaic of cropping; 14-forest mosaic/degraded forest.

KASC has an elevation range from 9 to 3,942 m above sea level (Fig. 1), with the majority (>90%) of the area lower than 2,000 m (Fig. 6b). Moreover, all the elevation gradients have <10% of their areas under the protection of NRs (Fig. 6b). Highlands in the western mountainous region of the study area are rarely protected and no NR was designed in the regions with elevation of >3,500 m. While, areas with the elevation between 2,000–3,500 m and <1,000 m have relatively higher proportions of conserved areas (Fig. 6b). Rapid expansion of the NR system was emerged in the areas with the elevation between 3,000–3,500 m (from 0.50% to 7.98%) during the past 20 years, all of which are covered by national NRs (Fig. 6b). More than half of the conserved areas within <1,500 m and 2,500–3,000 m are national NRs (Fig. 6b).

There are seventeen vegetation types in this area. Subtropical acid soil evergreen-broadleaved deciduous shrub forest, coppice and meadow (154,405.53 km^2^) is the most widely distributed vegetation, which is followed by tropical-subtropical limestone lianoid vegetation (82,220.62 km^2^), subtropical limestone broadleaf and mixed forest (75,606.40 km^2^), and subtropical-tropical evergreen conifer forest (66,104.67 km^2^) (Fig. 6c). The areas of all the other vegetation types are <40,000 km^2^. Generally, most of the vegetation types have <20% of their areas under protection, except for tropical semi-evergreen broadleaf monsoon forest and secondary vegetation (22.46%) and open water (28.36%) (Fig. 6c). Only 1.08% and 0.83% of the areas of tropical-subtropical dry shrub forest and grassland and tropical-subtropical triple cropping farmlands are protected, and subtropical evergreen sclerophyllous broadleaf forest and subtropical bamboo forest are even not covered by the NRs (Fig. 6c). No obvious increase of proportion of protection was detected in most vegetation types after 1990, although the coverage of NRs for open water and desert/none vegetation area were nearly doubled (from 15.24% to 28.36%) and quadrupled (from 3.44% to 14.23%, mainly contributed by newly established national NRs), respectively (Fig. 6c). There are eight vegetation types have >70% of their conserved areas covered by national NRs. Subtropical acid yellow soil broadleaf and mixed forest (100%) and tropical evergreen rain forest and secondary vegetation (96.19%) are well conserved by national NRs (Fig. 6c).

Fourteen landcover types occur in the karst area (Fig. 6d). Needleleaved evergreen forest (148,267.30 km^2^) has the largest range, which is followed by bush (129,121.82 km^2^), farmland (90,734.16 km^2^), and broadleaved deciduous forest (50,417.67 km^2^) (Fig. 6d). All the other landcover types have areas of <40,000 km^2^. Cities and towns has the highest protection proportion of 46.51%, followed by river (18.91%), lake (16.60%), bare land (13.39%), and broadleaved deciduous forest (10.37%) (Fig. 6d). The areas overlain with NRs of the other nine landcover types are relatively small (<10%) and the increase of protection proportion for each landcover type is limited after 1990 (Fig. 6d). Though lake and river have the highest increases of protection proportions (lake: 5.72%, from 10.88% in 1990 to 16.60% in 2010; river: 3.63%, from 15.28% in 1990 to 18.91% in 2010), they are mainly covered by provincial NRs (98.50% and 91.13%) (Fig. 6d). Meanwhile, 2.77% of the areas of bush and 2.17% of the areas of needleleaved evergreen forest were overlay with NR system, with 63.16% and 63.47% of them were under the conservation of national NRs (Fig. 6d). Nine landcover types even have more than half of their conserved areas covered by national NRs, with forest mosaic/degraded forest possessed the highest protection proportion (91.73%) (Fig. 6d).

Over 80% of the karst area was under relatively low HFP (20–40), although 40–60 of HFP covers nearly 15% of the total range (Fig. 6e). The results showed that NRs covered 32.28% of the areas with HFP >80, whereas the regions with lower human disturbance have protection proportions less than 10% (Fig. 6e). The increasing rates of protected proportion for all the HFP categories were quite low (0–2.23%), and no enlargement of NRs in the regions with HFP >80 was occurred (Fig. 6e). The areas with HFP <20 has 68.63% of its conserved area covered by national NRs, which is the highest proportion among the HFP gradients. While, all the NRs in the areas with HFP >80 are provincial NRs, with a quite small total area of 189 km^2^ (Fig. 6e).

## Discussion

Karst area in southwest China has a unique fauna and is rich in terrestrial vertebrates with a large number of endemic species. But, as human population increases rapidly in KASC in the recent decades, anthropogenic influence is expanding and the original vegetation is destroyed^32^. Furthermore, many species there are over-exploited as traditional medicines and foods^32^. Consequently, wild animals are faced with population decline, habitat fragmentation and loss. Biodiversity and habitat are fragile and, if destroyed, are difficult to recover. Although they have been slowly recovering since the National Wildlife Protection Law has been implemented since 1989, most of the wild animal resource cannot be harvested unless was approved by National Wildlife Management Authority^33^.

Efficiencies of biodiversity conservation for NR systems are often limited, because many of them are established based on social goals or economic roles rather than the importance on ecological characteristics^22^. Such an unbalanced status is showed in KASC, which is similar to that in the European Union^34^, Romania^18^, England^17^, and America^25^. Our study indicated that a number of counties with high species richness are under poor protection or even not covered by any NRs. These counties scatter in the entire karst area and conservation needs new NRs to fill up the existing gaps. In the regions with the richest animal species in Guizhou, national NRs only covered a limited area. As to Chongqing and Sichuan, although their areas of karst landscape are small, the rich biodiversity and specific fauna in the counties near the boundary of karst area need better conservation. In Guangxi and Yunnan, although their conservation systems are relatively developed, the distributions of their existing NRs need to be improved and more NRs are also needed.

When compared with the commonly used conservation target of 10% (top 10% biodiversity-rich areas and 10% of the areas for each biogeographic element)^35^, most NR systems tend to over-represent some type of areas and, oppositely, provide limited protection in other regions^25^. For example, 65.8% of English highlands have NR status, while quite a low percentage (3.5%) of lowlands in England is under protection^17^. Powell *et al*.^23^ revealed that only nine of the 23 life zones in Costa Rica are adequately represented, thereinto, three life zones contain >30% of their areas overlaying with NRs and 11 life zones are rarely protected. Similar situations among the 28 nature vegetation types in Brazilian Amazon are also reported^36^. Our results indicated that conservation representations are insufficient (<10%) in most of the ecoregions, elevation classes, vegetation types, landcover categories, and human disturbance intensity gradients in KASC. Although great efforts on nature conservation and NR system upgrading have been carried out during the past 20 years, growth rates of NR number and coverage in the karst area were still low, especially for the national NRs. Because of the lack of resource input, personnel and basic infrastructure for provincial NRs, efficacy of the NR system might be reduced because of the small area proportions of national NRs for many biogeographic categories. Besides, over-representation and under-representation are simultaneously occurred in this area. For example: Guizhou Plateau broadleaf and mixed forest and Yunnan Plateau subtropical evergreen forest occupy more than half of the total karst area, but no greater conservation efforts were conducted in these two ecoregions than those in the other ecoregions; the NRs within northern Indochina subtropical forest ecoregion and southeast China-Vietnam subtropical evergreen forest ecoregion need to be expanded because they support large number of species, although with relatively small areas; the protected proportions in the >2,000 m areas are generally higher than those in the lowlands, though highlands are limited in KASC and have low species richness; tropical semi-evergreen broadleaf monsoon forest and open water are rare in this area and with low species richness but over-represented, while four types of widely distributed vegetation and other three types of tropical and subtropical forests with rich SRs need severely consideration; For landcover and human disturbance categories, great conservation efforts focus on river, lake, city and town, suburbs, bare land, and other areas with high HFP indices, however, NRs in needleleaved evergreen forest, bush, farmland, mosaic of cropping, forest mosaic/degraded forest, and areas with low HFP (<40) should be expanded. Thus, standards and procedures used in conservation planning in KASC are problematic^37^ and specific targets for different biodiversity elements should be set according to their conservation value^38^. We suggest that expansion and adjustment of the NR system need a comprehensive integration of ecological, sociological, biogeographic, and economic conditions, including biodiversity pattern and diverse conservation capacities among different NR levels. To enhance NR system in this area, new NR construction as well as transformation of existing NRs should be conducted based on detailed information on the biodiversity patterns and its distributions traits among biogeographic elements.

Effective management of NRs extends beyond their establishment to many actions for their conservation and protection, which might lead many of them to be act as “paper NRs^27^,^28^”. There are a number of NRs below national level in this region, and NR coverages in some evergreen forests, medium elevation areas, highly disturbed regions (HFP >60, city and town), bare land, and open water are mostly contributed by provincial NRs. Although rapid increase of protection emerges in some areas, the effectiveness is usually not ensuring their ecological integrities^39^,^40^. As some of the provincial NRs have no recognizable boundary, land tenure, or routine monitoring schemes, wildlife management in these NRs needs to be improved and we should also promote these NRs to be national NRs. In addition, biodiversity in this region is severely threatened by habitat degradation caused by unsustainable resource utilization and uncontrolled increase of tourism^41^. We suggest that NR management should be standardized and NR selection procedures should be improved by focusing on not only endangered species but also the protection of their karstic habitats. However, it is obvious that establishing of NRs cannot be considered as the ending point of biodiversity conservation^4^.

As range map is a abstraction of species distribution but not real species occurrences everywhere^42^, large parts of their extents may be unsuitable for protection^3^,^43^. Thus, conservation problems can not be solved by NRs alone but should be considered in a broader framework of all local ecological and socio-economic plans^4^. We should integrate within-NR and off-NR managements, including the preservation of natural processes, controlling the increase of human disturbance on the ecosystems, stimulating the growth of local economy, and the development of local community and NR infrastructures^4^. Furthermore, as Gap analysis has one limitation that there is no explicit analysis on connectivity among species distribution ranges, biodiversity hotspots, and NRs, more detailed studies about this issue should be conducted before establishment of conservation plans and NR selection^20^. As southwestern karst area is an underdeveloped region in China, more attention should be put on the poverty alleviation to improve the livelihood of local people and more governmental and private funding are needed for the development of NR system. Besides, long-term biodiversity monitoring scheme should be implemented, and illegal hunting and the trades of wildlife resources should be forbidden for protecting the unique fauna in this area.

The data used in the study are of coarse scale. We only had the species information in county level due to the methods used in the past field expeditions when people had no GPS. This is a nature of many zoological records and conservation practices. However, we can still extract the distribution pattern of terrestrial vertebrates and assess the *in situ* conservation status in KASC based the available data. As the spatial and temporal resolutions of the data could significantly impact the results of such kind of research, there is a urgently need for more systematic field inventory surveys to obtain precise documents on the species locations, health and trends of their habitat changes. Since this study included all the counties with >10% of their areas are karst but, in some counties, the variations of species distribution between karst and non-karst landscapes may be quite obvious, we suggest that a detailed study on karst distributions and a robust map of karst landscape throughout the Chinese southwestern area could be valuable for its biodiversity conservation planning. Since this study used coarse data that is readily available, its usefulness could be extended to other regions to assess the gaps among species protection status, species abundance, and conservation efforts.

## Methods

### Study Area

Our study covers the whole karst area (all the counties with >10% of their areas are karst landscape) in the southwestern China according to Compiling Committee of Comprehensive Physical Regionalization in China^44^, thus limits for inclusion of data spanned 21° N through 30° N and 99° E through 113° E (Fig. 1). Area of karst landscape in this region is 541,234.23 km^2^, including 279 counties in Guangxi, Guizhou, Yunnan, Chongqing, Hubei, and Hunan^32^. Guangxi has about 120,000 km^2^ of karst landscape, which accounted for 60% of its total area, while nearly all of Guizhou and half of Yunnan are karst region^29^. Human population in the karst area was over 186 million in 2010, with a higher population density than the national average^32^. Unique flora is found on the calcareous soil in this area and its diverse vegetation types provide suitable habitats for many native species^29^. Well known endemic animal species are *Presbytis leucocephalus, Stachyris nonggangensis, Boiga guangxiensis*, etc., which have very small distribution ranges, whereas *Gecko gecko* is an indicator reptile of karst cliff habitat^29^.

### Data Collection

We collected the species occurrence data of amphibians, reptiles, birds, and mammals for each county to establish a terrestrial vertebrate distribution database at county level. We conducted intensive literature surveys particularly on zoological references, animal range atlases, wildlife checklists of nature reserves, and reports of field investigations in the study region^45^,^46^,^47^,^48^,^49^,^50^. We also extracted species distributions from 111,814 specimen collections in the museums of Institute of Zoology, Chinese Academy of Sciences (CAS), Chengdu Institute of Biology, CAS, Kunming Institute of Zoology, CAS, Guangxi Normal University, Guangxi Institute of Biology, and Guizhou Institute of Biology (Fig. 1). Meanwhile, we checked the dataset according to the Vertebrate Information System of China maintained by our research group^51^. We conducted field surveys in Nonggang National Nature Reserve, Dayaoshan National Nature Reserve in Guangxi, Maolan National Nature Reserve in Guizhou, Shilin Geological Park and Changhu Park in Yunnan for rapid biodiversity assessments (Fig. 1). In addition, we held consulting panel meetings during 2008–2010 and consulted with over sixty zoologists and wildlife managers from Ministry of Environmental Protection to review our database. Since karst regions contain a lot of caves and the field surveys and animal distribution records in the caves of the Chinese southwestern karst area were insufficient, this study did not include biodiversity and ecosystems of the caves.

To create a spatial dataset of NR distributions in this region, we used the polygons from the World Database on Protected Areas^52^ and digitized the available maps within the NR survey reports. Then, we got ecoregion rasters from Olson *et al*.^53^ and the elevation data was obtained from a digital elevation model (DEM, 90 m of resolution)^54^. Human footprint (HFP) data from Last of the Wild Data^55^, which ranges from 0–100 (higher numbers indicate higher human impacts), was considered as an index of human disturbance in this research. Landcover and vegetation type rasters were obtained from Global Landcover 2000^56^ and China Vegetation Database^57^. All of these spatial data were at the scale of 1 × 1 km, and we then reclassified DEM into 500 m bands and split HFP into <20, 20–40, 40–60, 60–80, >80 intervals (to detect the conservation efficiencies of NR system in each DEM or HFP gradient, see below).

### Analyses

We calculated the species richness (SR) of amphibians, reptiles, birds, and mammals for each county and mapped the SR spatial patterns of the four taxa across KASC at county level using ArcGIS 9.3 (ESRI, Redland, USA). To explain the importance of terrestrial vertebrate resources and biodiversity conservation in KASC to the conservation efforts at the national scale, we counted the numbers of species which are endemic to China, listed in the “Checklist of National Protected Wild Animal Species of Beneficial, with Important Economic Value and Scientific Value” (NPWAS-BES)^58^, the “Wild Animal Species with Matured Artificial Breeding Technology” (WAS-MABT)^59^, the “Economic Fauna” (EF)^60^,^61^, the “Terrestrial Vertebrates of Medicine Value” (TVMV)^62^, the “IUCN Red List of Threatened Species” (IUCN Red List)^63^, the “National Key Protected Wild Animal Species” (NKPWAS, Category I and Category II)^64^, the Appendix I and Appendix II of “Convention on International Trade in Endangered Species of Wild Fauna and Flora” (CITES)^65^, and the Appendix I and Appendix II of “Convention on Migratory Species” (CMS)^66^.

Considering the cost and the lack of funding of biodiversity conservation, conservation planning needs an identification of biodiversity hotspots and conservation priorities to support the most species at the least cost^1^. To explore the representation on local biodiversity of the NR system and search for their vulnerabilities, we processed a Gap analysis by overlaying the species richness map and NR coverage^19^. The hotspots were defined as the top 10% of counties in species richness of terrestrial vertebrates in this study, because these counties show high representativeness of amphibians, reptiles, birds, and mammals of KASC (77.3%, 82.4%, 83.5%, and 75.6%; see the Results). We focused on national and provincial NRs in these analyses, because their conservation effectiveness is higher and more reliable^39^,^40^. To address the SR patterns of terrestrial vertebrates among the parameters of ecoregion, DEM, HFP, landcover, and vegetation type, we counted the amphibian, reptile, bird, and mammal species that occurring in each type or category within each raster, respectively. Furthermore, to quantify the conservation status for the five biogeographical parameters, we first counted the cells for each type or category in the rasters. By following Iojã *et al*.^17^ and Oldfield *et al*.^18^, we counted the cells overlapping with national and provincial NRs within each type or category in each raster. Then, the proportion of area that under the protection of national and provincial NRs before and after 1990 (till 2010) for each category was calculated separately, and their difference was calculated as an index of increasing rate of protection. We also computed the area percentages of national NRs/(national NRs + provincial NRs) within all the variable categories.

### Ethic Statements

No experiment on live vertebrates was included in this study. All of our research was performed in accordance with relevant laws, guidelines, and regulations of China and under a permit issued by the Institute of Zoology, Chinese Academy of Sciences. All the spatial analyses were carried out in ArcGIS 9.3 (ESRI, Redland, USA) and no third party overlay was used with the mapping programme.

## Additional Information

**How to cite this article**: Luo, Z. *et al*. Conservation of Terrestrial Vertebrates in a Global Hotspot of Karst Area in Southwestern China. *Sci. Rep*. **6**, 25717; doi: 10.1038/srep25717 (2016).

## Figures and Tables

**Table 1:** Terrestrial vertebrate resources and their conservation importance in the karst area, southwestern China.

	Amphibian	Reptile	Bird	Mammal	Total
NPWAS-BES^a^	118	178	348	41	685
WAS-MABT^b^	2	1	4	4	11
EF^c^	3	0	189	66	258
TVMV^d^	7	27	31	21	86
IUCN^e^-CR^e1^	3	1	0	2	6
IUCN-EN^e2^	5	4	8	7	24
IUCN-VU^e3^	10	9	21	6	46
IUCN-NT^e4^	6	2	11	2	21
NKPWAS^f^-I^f1^	0	3	10	12	25
NKPWAS-II^f2^	4	5	96	17	122
CITES^g^-I^g1^	1	0	20	11	32
CITES-II^g2^	1	16	56	16	89
CMS^h^-I^h1^	0	0	119	0	119
CMS-II^h2^	0	0	135	36	171

The numbers mean species amount in each category for each taxa.

^a^Checklist of National Protected Wild Animal Species of Beneficial, with Important Economic Value and Scientific Value.

^b^Wild Animal Species with Matured Artificial Breeding Technology.

^c^Economic Fauna.

^d^Terrestrial Vertebrates of Medicine Value.

^e^IUCN Red List of Threatened Species; e1 critically endangered, e2 endangered, e3 vulnerable, e4 near threatened.

^f^National Key Protected Wild Animal Species; f1 Category I, f2 Category II.

^g^Convention on International Trade in Endangered Species of Wild Fauna and Flora; g1 Appendix I, g2 Appendix II.

^h^Convention on Migratory Species; h1 Appendix I, h2 Appendix II.
